# Assessing nursing information competence of nurses and identifying the associated influencing factors

**DOI:** 10.3389/fmed.2025.1646236

**Published:** 2025-11-13

**Authors:** Yun-xia Li, Xiao-wen Fan, Chen Huang, Hong-ying Pan

**Affiliations:** Nursing Department, Sir Run Run Shaw Hospital, Zhejiang University School of Medicine, Hangzhou, China

**Keywords:** nurses’ informatics competence, correlated variables, regression model, nurses’ nursing information competence, health information technology, competency assessment, nursing workforce

## Abstract

**Purpose:**

The objective of this study was to assess the level of nursing information competence (including computer competence, information competence, and comprehensive application competence dimensions) of nurses and determine the influencing factors related to nursing information competence.

**Background:**

The wide application of information technology in medical system can improve the quality of nursing service. At present, the evaluation results of nurses’ informatics competence are not consistent.

**Methods:**

This cross-sectional study survey was carried out in a tertiary hospital in Zhejiang, China, November 3 to November 9, 2023. Nurses who meet the inclusion and exclusion criteria were recruited through purposive sampling method.

**Results:**

This study involved 1,237 nurses. The median score of nurses’ informatics competence of 1,237 nurses was 98 (83,110), and the median score for each item was 2.88 (2.44, 3.24). The nursing information competence score were divided into three groups: high group (358, 28.94%), medium group (598, 48.34%), and low group (281, 22.72%), with score of 94 (interquartile range: 15), 71 (interquartile range: 14), 49 (interquartile range: 19). Eight variables strongly correlated with the total nursing information competence score were identified: (1) ability to adopt corresponding educational methods to disseminate information technology knowledge; (2) ability to effectively use clinical information according to work needs; (3) ability to evaluate the quantity and quality of information resources and adjust search strategies as needed to further obtain information; (4) ability to combine quality management tools to analyze and apply information; (5) ability to regularly collate medical record data using information systems and classify them by different purposes; (6) ability to select search strategies and effectively obtain information from the internet or electronic databases; (7) ability to clearly express problems encountered in information technology use; (8) familiarity with commonly used network terms. The prediction accuracy of Gradient Boosting regression and Linear regression models reached 0.829 and 0.836, respectively.

**Conclusion:**

This study shows that the informatics competence score of nursing staff is in the middle to upper level, and hospital managers can carry out more targeted and personalized training in the aforementioned 8 aspects.

## Introduction

The rapid development of information and communication technology (ICT) and its wide application in healthcare systems have brought colossal challenges to the improvement of nurses’ knowledge of nursing informatics in the medical field. Healthcare providers need basic computer skills, information competencies, and informatics knowledge to manage health information more effectively (1). Nurses’ informatics competence refers to the ability to integrate knowledge, skills, and attitudes toward the performance of various nursing information activities within the prescribed level of nursing practice (2). In the 1990s, the American Nurses Credentialing Center (ANCC) carried out the certification of nurses’ informatics competence (3). Nurses with better information competence can proficiently utilize the clinical file system, leading to improved work efficiency and more time available for patient care. Additionally, they can obtain evidence-based information, assist clinical decision-making, and deliver accurate, high-quality nursing for patients, so as to improve the quality of nursing service and ensure the safety of patients (4–6). A research (4) showed that most nursing staff consider informatics to be an important nursing competence (91.3%). At present, the ability of registered nurses in informatics and the factors related to informatics competence have been investigated both domestically and internationally. A survey (7) conducted in 14 hospitals in Palestine revealed that nurses exhibited lower levels of nursing information competence, especially in the area of information skills. In an analytical descriptive study (8), which surveyed 205 subjects, it was found that nursing information competence score was low and computer skills-related competence was at the lowest level. Multiple domestic studies (9–11) showed that nurses’ nursing information competencies are ranging from intermediate to advanced levels, whereas the results of nurses’ nursing information competencies were inconsistent.

In the era of digital healthcare, the importance of nurses’ informatics competence cannot be overstated. As frontline providers of patient care, nurses’ ability to proficiently apply information technology directly impacts clinical work efficiency, the accuracy of evidence-based decision-making, and ultimately the quality and safety of nursing services. Although numerous domestic studies have assessed nurses’ informatics competence, these studies primarily focus on describing competence levels or identifying general influencing factors (12). Against this backdrop, our hospital has established a unique nursing informatics management system characterized by vertical integration and flattening. Led by nurses specialized in informatics and centered on the Nursing Information Committee, this system aims to promote the specialization of nursing informatics practice. The uniqueness of this study lies in three aspects: first, it not only assesses the current level of informatics competence among nurses in our hospital and identifies key influencing factors, but also uses two regression models to verify the predictive effect of these factors, providing a quantitative basis for targeted training; second, it indirectly evaluates the effectiveness of the aforementioned hospital-specific informatics management system by analyzing the informatics competence level of nurses under this system—filling the gap in linking institutional management models to nurses’ competence outcomes. To clarify the research direction and address existing gaps, this study focused on the following specific research questions: (1) What is the level of nursing information competence (including computer competence, information competence, and comprehensive application competence dimensions) among nurses in our hospital? (2) Which variables have a strong correlation with the total score of nurses’ nursing information competence, i.e., what are the key influencing factors? (3) How effective are these key influencing factors in predicting the total score of nurses’ nursing information competence? Therefore, the purpose of this study was to assess the level of nursing information competence of nurses in our hospital, determine its associated influencing factors, and verify the predictive effect of these factors, so as to identify the educational and supporting needs of nurses in informatics.

## Methods

### Study participants

A total of 1,237 nurses from Sir Run Run Shaw Hospital School of Medicine Zhejiang University were selected as subjects from November 3 to November 9, 2023 by purposive sampling method. The study participants included nurses from the hospital across different age groups, work experience levels, job titles, and positions (see Table 1 “Demographic Characteristics of Nurses”). With a sample size of 1,237, the sample can fully represent the overall characteristics of the hospital’s nurse population and reduce sampling errors. Inclusion criteria were (1) having a nurse professional qualification certificate and (2) informed consent, voluntarily participating in this study. Exclusion criteria were (1) refresher nurses and (2) practice nurses.

**Table 1:** Demographic characteristics of nurses.

Variable	Group	Overall	Percentage (%)
Age	20–25	248	20.05
	26–30	378	30.56
	31–35	359	29.02
	36–40	147	11.88
	>40	105	8.49
Gender	Male	33	2.67
	Female	1,204	97.33
Work experience	0–1	117	9.46
	1–2	111	8.97
	3–5	221	17.87
	5–10	406	32.82
	10–20	297	24.01
	>20	85	6.87
Has participated in or is currently a member of the NIC	Yes	184	14.88
	No	1,053	85.13
Job title	Nurse	119	9.62
	Senior nurse	464	37.51
	Supervisor nurse	638	51.58
	Co-chief superintendent nurse	14	1.13
	Chief superintendent nurse	2	0.16
Post	Primary nurse	114	9.22
	Nursing team leader	852	68.88
	Practice teaching	169	13.66
	Head nurse	56	4.53
	Supervisor of nursing care	46	3.72
First degree	secondary	67	5.42
	College or vocational	54	4.37
	Bachelor	1,090	88.12
	Postgraduate	26	2.10
Final education or degree	Secondary	1	0.08
	College or vocational	5	0.40
	Bachelor	1,173	94.83
	Postgraduate/master degree or above	58	4.69
Whether there have been issues related to reporting nursing information in the last 5 years	Yes	61	4.93
	No	1,176	95.07
Whether any core journals related to nursing information have been published in the last 5 years	Yes	45	3.64
	No	1,192	96.36
Have you applied for software copyright or information-related utility model patents/national invention patents in the past 5 years	Yes	95	7.68
	No	1,142	92.32

### Study design and tools

This is an cross-sectional study. Demographic characteristics include age, gender, work experience, job title, post, educational background, whether a member of the Nursing Information Committee, etc. A 5-point Likert scale was used for scoring (0 = not competent to 4 = very competent), with a total score ranging from 0 to 136 (higher scores indicate stronger informatics competence). Prior to formal data collection, we re-evaluated the reliability and validity of the scale for the study population.

Reliability: The overall Cronbach’s *α* coefficient was 0.954, and the Cronbach’s α coefficients for the three dimensions (computer competence, information competence, and comprehensive application competence) were 0.881, 0.903, and 0.927, respectively—all greater than 0.8, indicating excellent internal consistency.

Validity: The KMO value was 0.956 (>0.7), and Bartlett’s test of sphericity was significant (*χ*^2^ = 12,458.76, *p* < 0.001), confirming suitability for exploratory factor analysis (EFA). Using principal component analysis with varimax rotation, EFA extracted five common factors—all with eigenvalues greater than 1 (eigenvalues: 11.23, 5.87, 3.42, 2.15, 1.89). The cumulative variance explained by these five factors was 67.617%, exceeding the recommended threshold of 60%, which indicates good construct validity.

Although EFA identified five factors, we retained the original three-dimensional framework of the scale (computer competence, information competence, comprehensive application competence). This decision was based on two considerations: (1) The five extracted factors were largely consistent with the original three dimensions—Factors 1 and 2 were sub-components of “computer competence,” Factors 3 and 4 belonged to “information competence,” and Factor 5 corresponded to “comprehensive application competence”; (2) Using the original three-dimensional structure ensured consistency with previous studies (9, 13).

### Data collection

Before distributing the electronic questionnaires, informed consent was obtained from all nurses via the online tool “Questionstar.” The first page of the questionnaire link contained a clear informed consent statement, which included details such as the study purpose, scope of data use, confidentiality measures, and the right to withdraw from the study at any time. A mandatory checkbox was set for nurses to confirm their consent—only after checking the box stating “I have read and understood the content of the informed consent and voluntarily participate in this study” could they access and complete the questionnaire. A total of 1,237 questionnaires were distributed, and 1,237 valid questionnaires were collected, resulting in an effective recovery rate of 100%. The online informed consent process was recorded in the “Questionstar” system to ensure traceability, which complies with the ethical requirements for electronic data collection.

### Statistical analysis

SPSS 25.0 was used for statistical analysis of the data. Two methods were adopted: (1) The Shapiro–Wilk test (suitable for small to medium sample sizes, *n* < 5,000), which evaluates the goodness of fit of the data to a normal distribution; (2) Visual inspection via Q–Q plots (quantile-quantile plots), which visually assess whether data points are close to the theoretical normal distribution curve. Qualitative data were presented as frequencies and percentages. Quantitative data that passed the normal distribution test (Shapiro–Wilk test, *p* > 0.05; Q–Q plots showing data points closely coinciding with the theoretical line) were described as mean ± standard deviation; those that did not conform to the normal distribution (Shapiro–Wilk test, *p* ≤ 0.05; Q–Q plots showing obvious deviation from the theoretical line) were described as median and interquartile range (IQR, expressed as P25, P75). Results were considered statistically significant at the level of *p* ≤ 0.05.

## Results

### Demographic characteristics

This study included 1,237 nurses, and all nurses were confirmed eligible. There were 33 males (2.67%) and 1,204 females (97.33%). Nurses aged from 26 to 30 years old accounted for 30.56%. Working years: 117 (9.46%) with 0–1 years, 111 (8.97%) with 1–2 years, 221 (17.87%) with 3–5 years, 406 (32.82%) with 5–10 years, 297 (24.01%) with 10–20 years, 85 (6.87%) with more than 20 years. Other information such as job title, post, and first degree are shown in Table 1.

### Nursing information competence

The median score of informatics competence of 1,237 nurses was 98 (83, 110), and the median score for each item was 2.88 (2.44, 3.24). The median of the total score of the computer ability dimension and the score of each item were 34 (27, 40) and 2.62 (2.08, 3.08), respectively. The median of the total score of the information competence dimension and the score of each item were 36 (30, 41) and 3 (2.5, 3.42), respectively. The median of the total score of the comprehensive application competence and the score of each item were 27 (23, 31) and 3 (2.56, 3.44), respectively, as shown in Table 2. Cluster analysis of the data from the 8 variables showed that nursing information competence can be roughly divided into three group: high group, medium group, and low group. The high group was skewed distribution. There were 358(28.94%) nurses in the high group, and the score of nursing information competence was 94 (interquartile range: 15). The medium group is skewed distribution. There were 598(48.34%) nurses in the medium group, and the score of nursing information competence was 71 (interquartile range: 14). The low group conformed to the normal distribution. There were 281 (22.72%) in the low group, and the score of nursing information competence was 49 (interquartile range: 19), as shown in Table 3.

**Table 2:** Nursing information competence level [Median (Lower Quartile, Upper Quartile)].

Variables	Total score	Average score of items
Total nursing information competence	98 (83, 110)	2.88 (2.44, 3.24)
Computer competence	34 (27, 40)	2.62 (2.08, 3.08)
Information competence	36 (30, 41)	3 (2.5, 3.42)
Comprehensive application competence	27 (23, 31)	3 (2.56, 3.44)

**Table 3:** Groups of nursing information competence level.

Group	Mean ± Standard deviation	Median (IQR)	Maximum value	Minimum value	Range	*k*–*s* test (p)
High	96.07 ± 12.98	94 (15)	136	72	64	0.000
Middle	71.03 ± 10.59	71 (14)	103	35	68	0.001
Low	48.94 ± 13.00	49 (19)	84	3	81	0.200

### Influencing factors of nursing information competence

**Figure 1:**
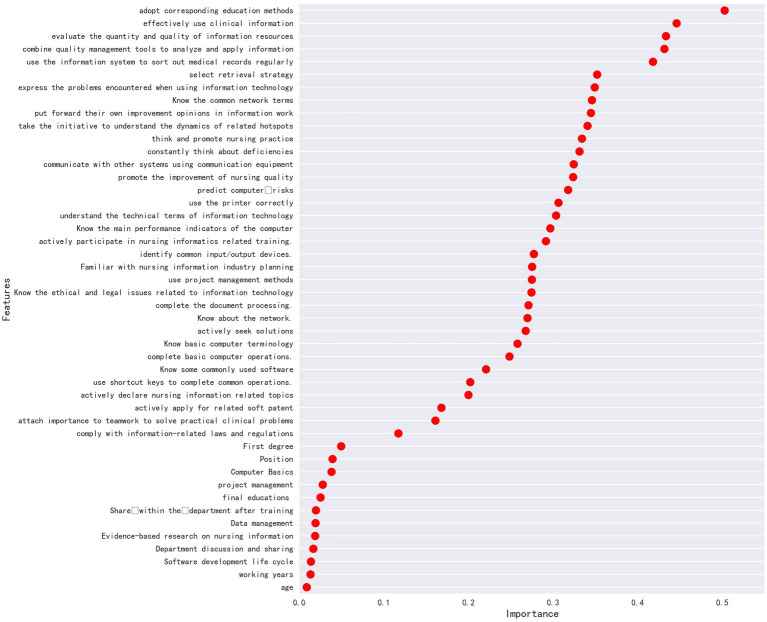
The correlation exploration method based mutual_info_regression.

Influencing factors were selected on the basis of the correlation exploration method based mutual_info_regression. There were 8 variables with a strong correlation with the total score of nursing information competence: (1) be able to adopt corresponding educational methods to disseminate knowledge of information technology; (2) effective use of clinical information according to the work needs; (3) be able to evaluate the quantity and quality of information resources, and adjust search strategies as needed to further obtain information; (4) be able to combine quality management tools to analyze and apply information; (5) be able to regularly collate medical record data using information systems and classify them according to different purposes; (6) be able to select the search strategies and effectively obtain information from the internet or electronic databases; (7) be able to clearly express the problems encountered in the use of information technology; (8) be familiar with commonly used network terms, as shown in Figure 1.

### Model evaluation

**Figure 2:**
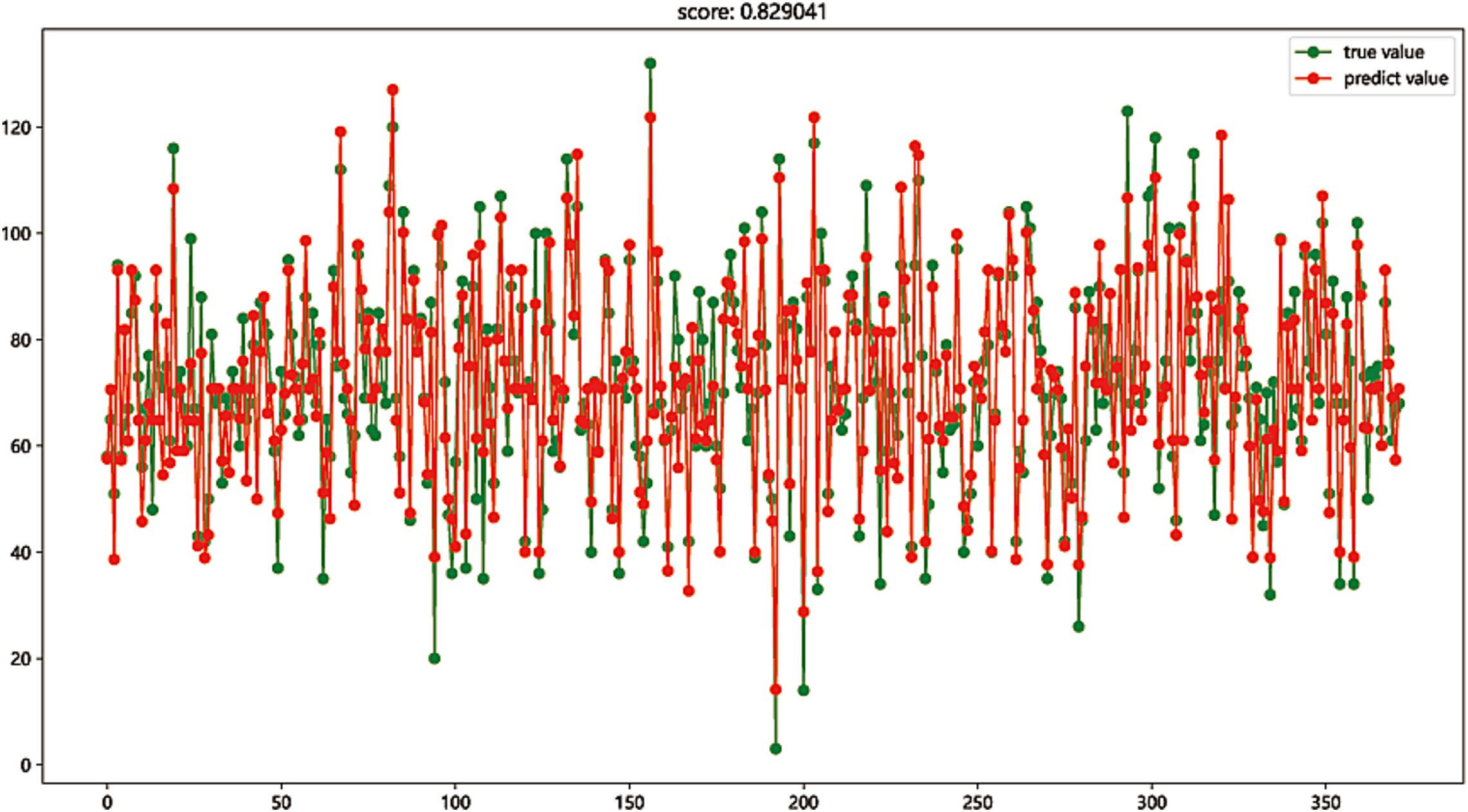
The regression model was established by Gradient Boosting regression (score: 0.829).

**Figure 3:**
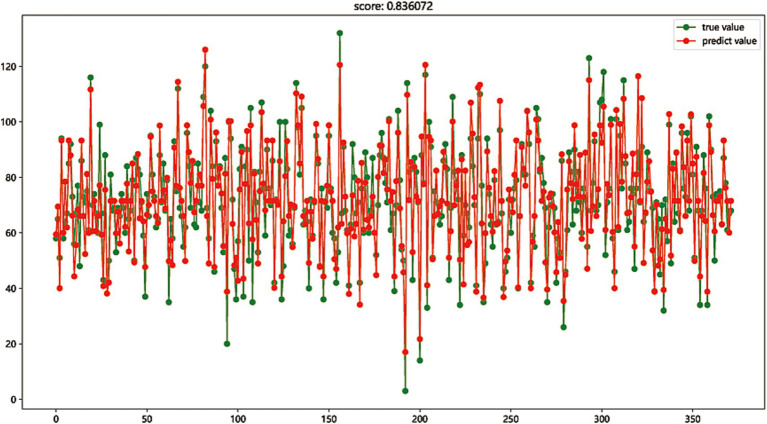
The regression model was established by Linear regression (score: 0.836).

The first eight most important variables were selected to perform regression fitting on the total score of nursing information competence to evaluate the expression competence of these eight important features to the total score of nursing information competence and the explanatory competence of the regression model. The regression model was established by Gradient Boosting regression and Linear regression. The prediction accuracy scores of the test set reached 0.829 and 0.836, respectively, showing good regression fitting performance. The results indicated that these 8 nursing information single scores have a high contribution to the comprehensive ability of nursing information competence, as shown in Figures 2, 3.

## Discussion

In the context of global digital healthcare transformation, nursing informatics competence has become a core competency for nurses, directly influencing clinical work efficiency, evidence-based decision-making accuracy, and the overall quality and safety of nursing services (2, 5). With the widespread application of information and communication technology (ICT) in healthcare systems, hospitals worldwide have accelerated the construction of nursing informatization, and the evaluation of nurses’ informatics competence and its influencing factors has become a key focus of nursing management research (1, 6). However, existing studies on nurses’ informatics competence show inconsistent results—some report low competence levels, particularly in information skills (7, 8), while others indicate intermediate to advanced levels (9–11). These discrepancies may stem from differences in research settings, sample characteristics, and assessment tools. Additionally, most prior studies have focused on describing competence levels or identifying general influencing factors, with limited use of quantitative models to verify the predictive value of key factors, and few have linked institutional informatization management models to nurses’ competence outcomes (12). Against this background, our study assessed the nursing informatics competence of 1,237 nurses from a tertiary hospital in Zhejiang, China, identified key influencing factors, and verified their predictive effects using two regression models.

### Nursing information competence analysis

The findings of the current study revealed that the median of informatics competence was 98, which was lower than other similar studies (8–10). The reason may be that the scales used were different. This study shows that the score of information competence dimension is at the highest level, followed by computer competence. Different from this study, another survey found that the subjects were most confident in the computer competence. The reason may be cultural differences. The United States attaches importance to informatics knowledge, skills development and data input capabilities. Otherwise, the results of this study reveal that approximately half of the nurses fall in the middle group, and over two-thirds of the nurses have nursing information competence scores in either the middle or high groups. It can be seen that nursing information competence of nurses is at a medium to advanced level, similar to other research results (11). Our hospital established the position of information nurse in 2017 (14). In addition, our hospital has mobilized all nursing staff to participate in information construction by constructing a nursing information management architecture, and carried out professional nursing information innovation and practice, thereby promoting the efficient development of the hospital’s nursing information industry and effectively improving the nursing information work environment. However, nursing information capabilities still need to be improved. It is necessary for nursing managers to provide nursing informatics-related education and training to clinical nurses. A research (4) suggests that training should consider the wider use of informatics in healthcare, rather than focusing solely on the technical skills required to operate specific technologies. This can be achieved through systematic and comprehensive educational programs and support resources to strengthen a culture that perceives informatics as an integral part of nursing practice rather than a technical skill.

### Influencing factors of nursing information competence

Two regression models with good fitting performance (Gradient Boosting regression and Linear regression; prediction accuracy: 0.829–0.836) confirmed 8 key variables correlated with informatics competence. Moreover, the hospital’s unique practices—including the establishment of an informatics nurse-led system in 2017, integration of electronic medical records (EMRs) with workflows, database search training, quality improvement projects requiring digital tools, and monthly IT newsletters—directly influenced these variables. The peer training mechanism led by informatics nurses enhanced nurses’ ability to disseminate information technology knowledge; the requirement for EMR data categorization improved their skills in organizing medical record data; and regular search training boosted their information retrieval and resource evaluation capabilities. These practices collectively strengthened the correlation between the 8 variables and competence, distinguishing our findings from those of studies lacking links to institutional practices. It shows that nurses who are able to adopt corresponding educational methods to disseminate knowledge of information technology have higher levels of nursing information competence. With the advancement of communication technology, the variety of education methods is increasing day by day. For example, the integration of information and communication technology with nurse training has led to the development of nurse education methods from initial multimedia courseware and teaching videos to the design and operation of current systems and teaching software (15). Nurses who are familiar with courseware and video production are likely to demonstrate a higher proficiency in utilizing electronic tools. According to the national survey of Canadian nurses who incorporate Digital health technology into their practice, nurses who frequently utilize electronic tools exhibit higher levels of confident than those who do not use electronic tools (16).

The results of this study indicate that nurses who are able to select the search strategies and obtain effective information from the internet or electronic databases, who are able to evaluate the quantity and quality of information resources and adjust search strategies as needed to further obtain information, and who are able to effectively use clinical information based on their work requirements demonstrate higher nursing information abilities. A survey (17) on the information competence and needs of nursing undergraduate students shows that their information acquisition ability needs to be improved, with only 22.32% being able to use search strategies to search for nursing information. The vast majority of nurses in this study have a bachelor’s degree, therefore, education and training on nurse retrieval strategies can be provided to improve their nursing information skills. In nursing, nurses are primarily engaged in clinical work, and the problems encountered in clinical practice often are important clinical information. To solve the problems above, nurses are expected to possess various skills, including computer skills, software management, familiarity with nursing information systems, database-related nursing, and effective web search methods to obtain necessary nursing information (6). Nurses with nursing information skills know how to effectively discover, evaluate, and use information, thereby improving the quality of information processing (1). Therefore, managers and relevant departments should conduct training for nursing staff in stages. After laying a solid foundation, it is essential to strengthen the training of nursing staff’s competence of information utilization and management, which will foster the rapid development of hospital nursing information level, ensuring effective and safe nursing practices while elevating the quality of clinical nursing (9).

We also found that nurses who are able to combine quality management tools to analyze and apply information have higher nursing information abilities. The implementation of the quality management project involves the use of Document retrieval, electronic forms, etc. The nurses involved in the project need to have computer skills, the use of statistical software, retrieval database skills, etc. Therefore, nurses who use quality management tools to analyze clinical information may have a higher competence of nursing information. This study indicates that the ability of regularly organizing medical record data using information systems and classify them according to different purposes is a influencing factor of nursing information competence. In a survey of 42 graduates’ self-awareness regarding their information technology skills, students expressed the highest level of confidence in basic computer skills, but provided the lowest evaluation of their ability to collate nursing documents (18). Considering the importance of regularly organizing medical record data using information systems, a study (12) suggests that new nurses should undergo pre-job training and assessment related to computer skills operation and nursing information systems, such as electronic medical record systems. Also, for already employed nurses, continuous computer-related training programs and computer operations included in daily skill assessments are recommended.

The innovation and development of information technology present a challenge for nurses. Our study found that being able to clearly express the problems encountered in the use of information technology is positively correlated with nurses’ nursing information competence. Nurses can understand the problems encountered when using information technology and learn skills through network search, database retrieval, and targeted learning to solve problems. The process of problem-solving is a process of improving nursing information skills. In addition, nurses who are familiar with commonly used network terms have a higher level of nursing information competence. Familiar with commonly used network terminology means that nurses are more acquainted to computers, and are more likely to have advantages in basic computer knowledge and skills as well as computer applications, thus possessing a higher level of nursing information competence (19).

### Relevance to clinical practice

The application of various information technologies has brought about significant changes in the working environment of nurses, which requires us to have high nursing information competence in order to better adapt to the new working environment and be competent in nursing work in the new era. The level of nurse nursing information competence directly affects the development degree of nursing informatization. This study has some implications for clinical practice. This study shows that the nursing information competence level of nursing staff is in the middle to upper level, this suggests that managers should pay attention to the training of medical staff, including not only clinical knowledge, but also the training of nursing information competence. For the content of nursing information competence training, first of all, it is necessary to educate nurses how to effectively obtain clinical information through retrieval strategies, to evaluate the quality of these clinical information resources, and to make effective use of them. Secondly, nurses should master the educational methods of disseminating information knowledge. Then, it is necessary to train nurses on how to efficiently organize medical records and classify them using nursing information systems. Finally, training can be carried out from common network terms and how to effectively express the problems encountered when using information technology.

## Limitations

The 1,237 participants in this study were recruited exclusively from a single tertiary Grade A general hospital in Zhejiang Province. Additionally, the hospital’s unique institutional informatization management model may overstate the competence level of the participants, thereby reducing the external validity of the study. Second, although 8 competence-related variables were identified through mutual information correlation analysis, the underlying mechanisms were not explored. This not only prevents the establishment of a causal relationship between these factors and competence but also fails to capture temporal changes in competence, thus limiting the evidence for the long-term effectiveness of interventions. Furthermore, no in-depth subgroup analysis was conducted for nurses with different job roles, educational backgrounds, or work experience. This makes it impossible to clarify the specific effects of these 8 factors on different subgroups, thereby reducing the practical applicability of the training recommendations proposed in the study.

## Conclusion

This study shows that the nursing information competence score of nursing staff is in the middle to upper level, and regression analysis results reveal that 8 influencing factors are the main factors affecting nursing information competence of nursing staff, which remind hospital managers and relevant departments to carry out more targeted and personalized training in the aforementioned 8 aspects. Training or education should involve instruction in computer skills, evaluation of information resources, retrieval methods, and effective utilization of information.

These findings have multifaceted positive impacts on the relevant field. Our research findings support the advancement of digital health and the improvement of public health service quality, and provide a replicable management model and quantitative evaluation tool for hospital nursing informatization. Furthermore, they enrich the evidence base for research on nursing informatics competence and offer guidance for curriculum optimization. Additionally, they help nurses identify competence gaps and enhance their professional competitiveness. Collectively, these contributions promote the development of nursing informatization.

## Data Availability

The raw data supporting the conclusions of this article will be made available by the authors, without undue reservation.
